# Interventions for Early-Stage Pericoronitis: Systematic Review of Randomized Clinical Trials

**DOI:** 10.3390/antibiotics11010071

**Published:** 2022-01-08

**Authors:** Tânia Oppido Schalch, Ana Luiza Cabrera Martimbianco, Marcela Leticia Leal Gonçalves, Lara Jansiski Motta, Elaine Marcilio Santos, Rebeca Boltes Cecatto, Sandra Kalil Bussadori, Anna Carolina Ratto Tempestini Horliana

**Affiliations:** 1 Postgraduation Program in Biophotonics Applied to Health Sciences, Universidade Nove de Julho (UNINOVE), São Paulo 01504-000, SP, Brazil; taniaschalch@gmail.com (T.O.S.); larajmotta@terra.com.br (L.J.M.); rebeca.boltes@gmail.com (R.B.C.); sandra.skb@gmail.com (S.K.B.); 2 Postgraduation Program in Health and Environment, Universidade Metropolitana de Santos (UNIMES), Santos 11045-002, SP, Brazil; analuizacabrera@hotmail.com (A.L.C.M.); marcelalleal@hotmail.com (M.L.L.G.); elaine.marcilio@unimes.br (E.M.S.)

**Keywords:** pericoronitis, treatment, systematic review

## Abstract

Background: To investigate the efficacy and safety of interventions for early stage pericoronitis. Methods: We searched for randomized controlled trials (RCTs) in databases from inception to July 2020, without language restriction. RCTs assessing adolescents and adults were included. Results: Seven RCT with clinical diversity were included, so, it was not possible to conduct meta-analyses. Individual study data showed an improvement in oral health quality of life in favor of topical benzydamine versus diclofenac capsule (Mean difference (MD) −1.10, 95% Confidence interval (CI) −1.85 to −0.35), and no difference between topical benzydamine and flurbiprofen capsule (MD −0.55 95% CI −1.18 to 0.0). There was no difference between diclofenac and flurbiprofen capsules (MD 0.55, 95% CI −0.29 to 1.39). An imprecise estimate of effects was found for all outcomes, considering (i) oral versus topic pharmacological treatment, (ii) different oral pharmacological treatments, (iii) pharmacological treatment associated with laser versus placebo laser, (iv) pharmacological treatment associated with different mouthwashes, and (v) conventional treatment associated to antimicrobial photodynamic therapy versus conventional treatment, with low to very low certainty of evidence. Conclusions: Until future well-designed studies can be conducted, the clinical decision for early stage pericoronitis should be guided by individual characteristics, settings and financial aspects.

## 1. Introduction

Pericoronitis is the common term used to describe the inflammation of soft tissues around the dental crown in a semi-erupted lower third molar [1]. The pseudo-pocket formed around the third molar accumulates bacterial plaque underneath the soft tissue cap, predisposing to inflammatory complications [2,3]. Usually, patients with early stage pericoronitis report: pain, intra-oral swelling, redness, mucosal ulceration, and loss of function [4]. Its cure is easy, quick, cheap, and no need for systemic antibiotics if detected early and appropriately treated [3]. Proper treatment of the initial phase is the local therapy over antibiotic prescribing [3]. Antibiotics should be reserved for severe cases where the spread of infection with systemic symptoms are present [5] because of the risk of developing resistance [6]. However, a large proportion of dentists routinely prescribe unnecessary antibiotics for pericoronitis [7]. The problem is the lack of evidence-based standardized treatment for initial pericoronitis [3].

It is well known that the evolution of the initial condition causes lymphadenopathy, fever, malaise, palatoglossal arch asymmetry, difficulty swallowing and trismus, indicating a more severe course of this condition [3] and may lead to a life-threatening condition called Ludwig’s angina [8]. Pericoronitis complications resulting in severe emergencies and must be treated in a hospital, with antibiotic cover. Unfortunately, antibiotics were prescribed to more than half of patients with pericoronitis, and, pericoronitis was among the top two in the frequency of antibiotic use [3]. The problem is the lack of evidence-based standardized treatment for initial pericoronitis, and evidence-based recommendations for its condition is not available until now. To date, there are no systematic reviews that have evaluated the best option treatment for initial pericoronitis, and it remains uncertain due to the lack of evidence. Therefore, the purpose of this systematic review was to investigate the efficacy and safety of treatments for pericoronitis in adolescents and adults to prevent its complications, reduce the use of antibiotics and antimicrobial resistance development.

## 2. Results

### 2.1. Search Results

**Figure 1:**
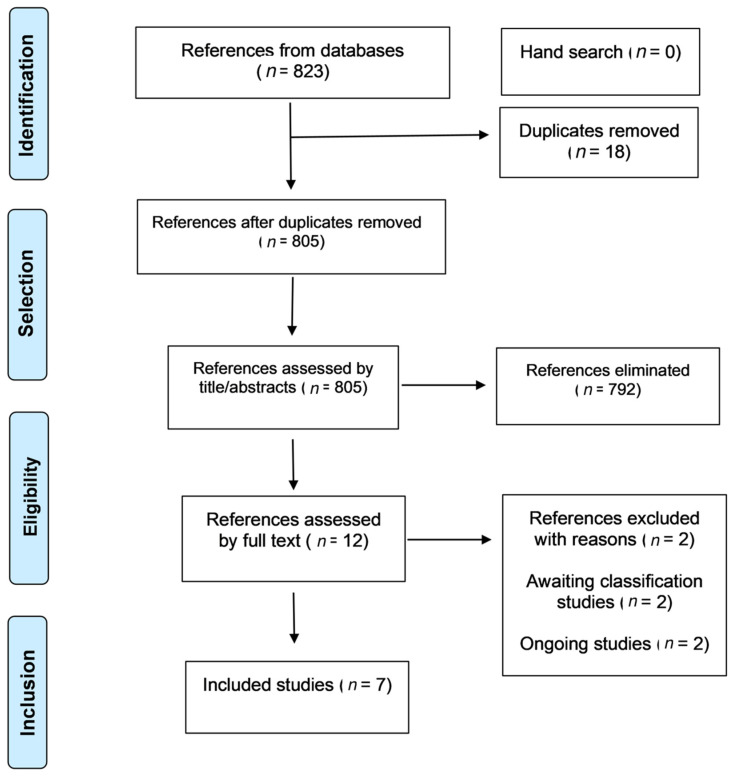
PRISMA flowchart of the study selection process.

The search strategies retrieved 823 references: 623 on MEDLINE, 23 on Cochrane Library, 78 on Embase, 74 on LILACS and BBO, six on Clinicaltrials.gov (www.clinicaltrials.gov, accessed on 1 January 2022), 19 on WHO/ICTRP and none on Opengrey. No additional record was identified through a hand search. Eighteen duplicate references were removed, and titles and abstracts screened 792 references. Of these, 13 studies were analyzed in full text, and two of them were excluded [8,9] due to combined interventions in comparison groups that did not fulfil the eligibility criteria. In addition, two studies [10,11] were classified as awaiting classification, one [10] because it was not clear what type of pharmacological intervention was administered and if both groups received the same co-intervention. Regarding the other study [11], we found neither the abstract nor the full-text paper. Both study authors were contacted by email, and there was no response until the final of this review. Two ongoing RCTs were found (NCT03919942 and NCT03576105). Thus, we included seven RCTs [12,13,14,15,16,17,18] in this systematic review. The flowchart of the search and screening of studies for the present review is shown in Figure 1 presents the flowchart of the study selection process. 

### 2.2. Characteristics of the Included Studies

This systematic review included seven RCTs conducted in the Middle East, three from Turkey [12,13,14], 1 in Saudi Arabia [15] and 1 in Iran [16]. One study was performed in England [17]. One study [18] did not report the country that it was conducted. Two studies [12,15] registered their study protocol. Only one study [15] reported following CONSORT [19] to conduct the study.

For the inclusion criteria, most of the included studies considered participants presenting pain [12,15,16], localized swelling [12,15,16], trismus [15,18], lymphadenopathy [13,16], recurrent infections [15] and malaise [16].

Participants were excluded if they were smokers [12,13,14,16], allergic to medications [12,14,16], using systemic analgesics or antibiotics in the last 3 days [12], 1 month [13] or 3 months [14,15,16,17] before the study. Some studies excluded pregnant and breastfeeding woman [13,14,15,16,17], systemic diseases [13,14,16] signals of severe pericoronitis, temperature, dysphagia, trismus, facial swelling, lymphadenitis, or malaise [12,15], local diseases, i.e., periodontal disease [14,17], a disease that is likely to influence submandibular lymph nodes such as sinusitis, upper breath respiratory tract infection, aphthous lesions, and herpes infection [13].

Included studies assessed the outcomes, evaluated between 3 days and 14 days after treatment. Only three RCTs [13,15,16] reported financial support sources. The included studies and their main characteristics are shown in Table 1.

### 2.3. Methodological Quality Assessment

**Figure 2:**
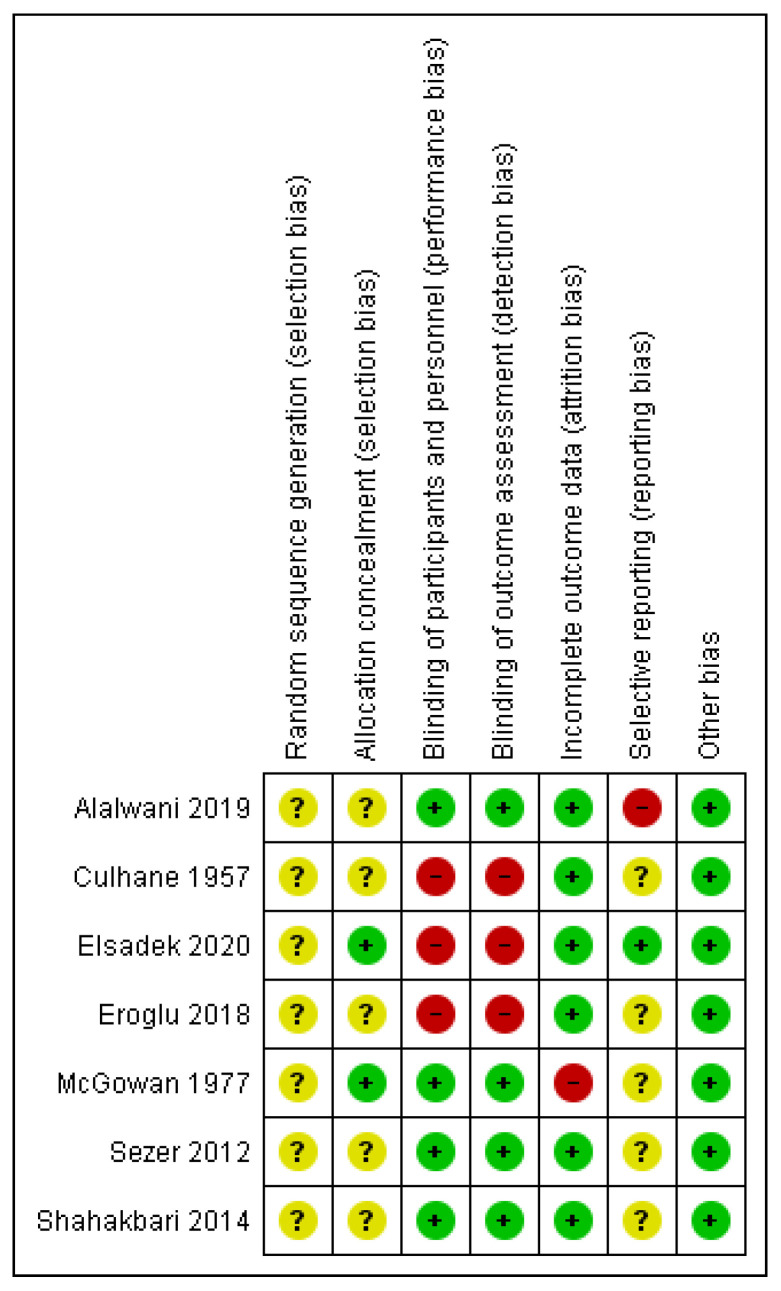
Summary of the risk of bias of included trials. (+) = low risk of bias; (?) = unclear risk of bias; (−) = high risk of bias.

The methodological quality assessment for each risk of bias item for each included study was summarized in Figure 2. We planned to assess blinding and incomplete outcome data domains by outcome level. However, none included studies have evaluated major adverse events. All studies showed an unclear risk of bias regarding the random sequence generation, as they presented insufficient information about randomization. Regarding the allocation concealment, only two studies [15,17] were classified as low risk of bias. Three studies [13,15,18] showed a high risk of bias on blinding of participants, personnel, and outcome assessors. Only one study [15] presented a high risk on incomplete outcome data due to a high rate of losses (27%) with no reason. Only two studies [12,15] presented protocol numbers of the clinical trial registration database (GH062254 and NCT03745599, respectively). However, one study [12] was classified as having a high risk of bias on selective reporting since the analysis of an important outcome (pain) was not mentioned in the study. The reasons for each judgment were detailed in Table S1.

### 2.4. Effects of Intervention

Given the clinical heterogeneity between included studies, it was not possible to group the results in meta-analyses. Thus, when numerical data were available, we calculated the estimated effects for the assessed outcomes of each comparison using Revman 5.4.1 [20].

#### 2.4.1. Comparison 1. Pharmacological Treatment: Oral vs. Topic

One RCT [12] (40 participants) compared diclofenac capsule (associated to a placebo spray) with topical benzydamine (associated to placebo capsule) and evaluated the following outcomes immediately after seven days of treatment:

- Any adverse events: diclofenac group presented lesser adverse events (gastrointestinal symptoms) than benzydamine (oral numbness and taste alterations), but there was an imprecise estimate of effect with a wide confidence interval, small sample size and reduced number of events (2/20 versus 11/20; Risk ratio (RR) 0.19 95% CI 0.05 to 0.72, *p* = 0.01).
- Quality of life (OHQoL questionnaire): better quality of life in favors of benzydamine (MD −1.10 points, 95% CI −1.85 to −0.35, *p* = 0.004).

The same RCT [12] also compared flurbiprofen capsule (associated to a placebo spray) with topical benzydamine (associated to placebo capsule) after seven days of treatment:

- Any adverse events: flurbiprofen group presented lesser adverse events than benzydamine, but there was an imprecise estimate of effect with a wide confidence interval, small sample size and reduced number of events (4/20 versus 11/20; Risk ratio (RR) 0.36 95% CI 0.14 to 0.95, *p* = 0.04).
- Quality of life (OHQoL questionnaire): no difference between groups (MD −0.55 points, 95% CI −1.18 to 0.08, *p* = 0.09).

Another RCT [18] (12 participants) compared penicillin with iodoform gauze drain associated with hot saline irrigations and reported fewer adverse events in the penicillin group (3 participants with gastric distress complaints) compared to no event in the iodoform group after six days of treatment. However, an uncertain estimated effect was due to the wide confidence interval and the lower number of participants and events (RR 7.00, 95% CI 0.44 to 111.91, *p* = 0.17). 

#### 2.4.2. Comparison 2. Different Oral Pharmacological Treatments

One RCT [12] (40 participants) compared diclofenac with flurbiprofen and evaluated the following outcomes immediately after seven days of treatment:

- Any adverse events: two participants in the diclofenac group and four in the flurbiprofen group reported gastrointestinal symptoms. The estimated effects are imprecise with a wide confidence interval and reduced number of events (2/20 versus 4/20; Risk ratio (RR) 0.50 95% Confidence interval (CI) 0.10 to 2.43, *p* = 0.39).
- Quality of life (OHQoL questionnaire): no difference was observed between groups (Mean difference (MD) 0.55 points, 95% CI −0.29 to 1.39, *p* = 0.20).

Another RTC [17] (31 participants) compared metronidazole versus phenoxymethylpenicillin and evaluated the following outcomes immediately after five days of treatment:

- Pain: lesser metronidazole participants presented pain when compared to phenoxymethylpenicillin. The estimated effect seems to show no difference between groups, but these are imprecise due to the wide confidence interval and the reduced number of participants and events (2/13 versus 1/18; Risk ratio (RR) 2.77 95% CI 0.28 to 27.4, *p* = 0.38).
- Trismus: the authors reported a final mean open mouth of 39.8 mm in the metronidazole group compared to 43 mm in the phenoxymethylpenicillin group (*p* > 0.05). It was not possible to calculate the mean difference because no standard deviation was provided.

#### 2.4.3. Comparison 3. Conventional Treatment Associated with Antimicrobial Photodynamic Therapy (aPDT) vs. Conventional Treatment 

One RCT [15] (59 participants) compared a conventional treatment (debridement, irrigation with warm saline, antiseptic, ibuprofen and instructed on oral hygiene care) associated with aPDT versus only conventional treatment. This study evaluated the following outcomes immediately after 14 days of treatment:

- Pain: the estimated effect showed a significant difference in the visual analogue scale favoring conventional treatment (MD 0.40 points 95% CI 0.19 to 0.61, *p* = 0.0002), but the reduction on the visual analogue scale (0.4 points) was not clinically relevant.
- Reduction of pro-inflammatory cytokines: the levels of interleukin 6 (IL-6) presented no difference between groups (MD 2.00 pg/mL 95% CI −10.72 to 6.72, *p* = 0.65), however, the tumor necrosis factor α (TNF-α) showed a significant reduction in favors of aPDT group (MD −128.00 pg/mL 95% CI −185.47 to −70.53, *p* < 0.0001), with an imprecise confidence interval.
- Microbiological assessment: there was a significant reduction in microbiological counts for both *Porphyromonas gingivalis* (MD −2.72 CFU/mL 95% CI −3.90 to −1.54, *p* < 0.00001) and *Tannerella forsythia* (MD −0.98 CFU/mL 95% CI −1.76 to −0.20, *p* = 0.01) in favors of aPDT group.
- Any adverse events: none of the participants presented any adverse events related to the interventions.

Another RCT [13] (40 participants) compared amoxicillin associated with antimicrobial aPDT versus amoxicillin alone and evaluated the following outcomes immediately after three days of treatment:

- Pain: The authors reported no difference between the two groups (*p* = 0.859). It was impossible to calculate the mean difference because the exact mean value was provided only on a graph (approximately 2,3 for both groups). Standard deviation was not provided.
- Any adverse events: None of the participants presented any adverse events related to the interventions.

#### 2.4.4. Comparison 4. Pharmacological Treatment Associated with Laser versus Placebo Laser

One RCT [14] assessed pharmacological treatment (amoxicillin trihydrate/potassium clavulanate, acetaminophen and chlorhexidine 0.12%) as co-interventions, associated with different types of laser:

- 1064-nm Nd: YAG versus placebo laser (40 participants): no difference was observed between groups on the OHQoL questionnaire (MD 1.50 points, 95% CI −2.31 to 5.31), pain reduction (MD 3.50 points 95% CI −9.79 to 16.79), and trismus (MD 1.50 mm, 95% CI −0.41 to 3.41). However, there were wide confidence intervals, and these estimated effects were imprecise.
- 808-nm diode (GaAlAs) versus placebo laser (40 participants): no difference was observed between groups on the OHQoL questionnaire (MD 2.30 points, 95% CI −0.60 to 5.20), pain reduction (MD −7.70 points 95% CI −17.97 to 3.97), and trismus (MD 0.15 mm, 95% CI −2.04 to 2.34). However, there were wide confidence intervals, and these estimated effects were imprecise.
- 660-nm diode versus placebo laser (40 participants): no difference was observed between groups on the OHQoL questionnaire (MD 0.25, 95% CI −2.24 to 2.74), pain reduction (MD −0.75 points 95% CI −12.30 to 10.8), and trismus (MD 1.55 mm, 95% CI −0.89 to 3.99). However, there were wide confidence intervals, and these estimated effects were imprecise.

#### 2.4.5. Comparison 5. Pharmacological Treatment Associated with Different Mouthwashes

One RCT [16] (97 participants) assessed the combined use of oral amoxicillin associated with green tea 5% versus amoxicillin associated with chlorhexidine 0.12% mouthwashes. The following outcomes were evaluated after seven days of treatment:

- Pain: There was no difference between groups, but these results are imprecise due to a wide confidence interval (MD −1.81 points, 95% CI −3.97 to 0.35).
- Trismus: There is no difference between green tea and chlorhexidine (MD 0.87 mm, 95% CI −0.23 to 1.97).

No subgroup nor sensitivity analyses were possible to be performed.

#### 2.4.6. Assessment of the Certainty of the Evidence

The certainty of the body of the evidence was classified as low to very low, according to the GRADE approach, for all primary outcomes of the most clinically relevant comparison: conventional treatment associated with antimicrobial photodynamic therapy (aPDT) versus conventional treatment. We downgraded this evidence to two levels due to methodological limitations and two due to imprecision (small sample size, single study, and wide confidence interval). The evidence is very uncertain about the effect of conventional treatment associated with aPDT on pain relief and reduction of pro-inflammatory cytokines (very low certainty) compared to conventional treatment alone. For microbiological counts (low certainty), the confidence in the effect estimate is limited (the true effect may be substantially different from the estimate of the effect). Thus, we have very little confidence in the effect estimate. The Summary of Findings table (SoF table) was presented in Table S2.

## 3. Discussion

There is no evidence-based standard of care for the treatment of initial pericoronitis [5]. As a result, a large proportion of dentists routinely prescribe unnecessary antibiotics [7] contributing to global antimicrobial resistance. Moreover, pericoronitis inadequately treated on a daily basis [3] can evolve to a life-threatening condition. Thus, it is important to summarize the available evidence-based standardized treatment to reduce its impact on patients’ quality of life, healthcare costs, and antimicrobial resistance development [3].

This systematic review assessed the efficacy and safety of treatments for pericoronitis. Seven randomized clinical trials with a total of 359 participants were identified. The methodologies used and the treatments evaluated were completely different among the included studies, making it challenging to infer the efficacy and safety of the treatments tested. Therefore, the certainty of the evidence for the main comparison was classified as low to very low according to the GRADE approach, which characterizes uncertain confidence in the results.

Pain is one of the main complaints reported by participants with pericoronitis. Of the seven studies found, five evaluated this outcome [13,14,15,16,17]. Among these, only one study [15] showed an improvement in pain improvement between treatments, favoring conventional treatment when compared with aPDT. However, this statistical improvement did not represent clinical relevance, suggesting that the conventional treatment adopted may not reflect practical advantages in routine use. The other studies that addressed pain [13,14,16,17] showed no difference between treatments but a wide confidence interval and reduced sample size and number of events, making the results imprecise.

The difficulty of mouth opening (trismus) has been assessed in three studies [14,16,17]. One study [17] compares metronidazole with phenoxymethylpenicillin and found no difference between groups. The use of antibiotics should always be weighted due to their already known adverse effects and bacterial resistance. Antibiotic prescribing should be reserved for severe conditions. A review [3] of antibiotic prescribing among dentists for pericoronitis shows an unnecessary use of this medication. They also applied a questionary which revealed almost 75% of dentists prescribed antibiotics for pericoronitis. They were given to more than half of the patients with pericoronitis. It is well known that the use of antibiotics must be reserved for severe cases of pericoronitis (systemic response or spread of infection) [3]. The study shows that early diagnosis and appropriate treatment of pericoronitis do not require antibiotic prescription. Antibiotics must be prescribed at the appropriate dose (minimum inhibitory concentration) and for the necessary length of time [3]. Commonly, the most prescribed antibiotics are amoxicillin or metronidazole. In severe cases, the frequency or dose can be increased [3], or consideration should be given to using both amoxicillin and metronidazole. For allergic patients, erythromycin may be used [9]. Patients with a swollen floor of the mouth, significant trismus, or difficult breathing must be transferred to the hospital [3].

In the initial phase of pericoronitis, without suppuration, the administration of these drugs should be avoided. Thus, alternative therapies with no side effects should be tested. In two studies [14,16] using different lasers versus placebo and chlorhexidine mouthwash versus green tea mouthwash, there was no difference between groups regarding mouth opening improvement. Green tea can be a product challenging to access in certain countries, which would make it difficult to use for participants with pericoronitis in specific locations.

Pericoronitis symptoms impact the quality of life of the individual, which was evaluated in only two studies [12,14]. One study [12] compared diclofenac versus flurbiprofen and flurbiprofen versus benzydamine topical spray, no differences were found between the groups regarding the quality of life, but these results are imprecise due to the wide confidence intervals found. When comparing diclofenac versus topical benzydamine spray, a better quality of life was noted favoring topical benzydamine. This result may be due to the almost immediate effect of the topical anesthetic of benzydamine, which would ensure some comfort for participants. Oral anti-inflammatory drugs need to find exact prescriptions since, in addition to gastric disorders, they may aggravate hypertension, renal and cardiac diseases, which reinforces the importance of alternative treatments for pericoronitis. Despite improving the oral health quality of life, the topical anesthetic presents quick action, which forces the patient to use it many times a day, exposing them to the toxicity of the topic anesthetic. The other study [14], comparing several types of lasers, found no significant difference in the quality of life in any group. However, these results are not consistent since the confidence intervals found were wide. The anti-inflammatory action of low-power laser is already well established, but irradiation parameters should be better investigated to offer effective treatment protocols for pericoronitis.

Only one study [15] conducted a microbiological and inflammatory cytokine analysis before and after the proposed treatments. The group submitted to aPDT showed a greater reduction of *P. gingivalis* and *T. forshytia*, as well as TNF-α, when compared to conventional treatment. This difference between the groups was significant, but the confidence interval when the TNF-α was analyzed was wide, which does not guarantee the accuracy of the result. However, when *P. gingivalis* and *T. forshytia* were analyzed, can reduce, or have no effect. Therefore, despite the inconclusive results regarding aPDT in pericoronitis, such therapy should be more investigated by its tested antimicrobial efficacy, absence of bacterial resistance, accessibility to performance, and low cost, making this treatment an interesting alternative for initial treatment pericoronitis [3]. For these reasons, the comparison that adds aPDT with conventional treatment was considered clinically relevant to have the certainty of the evidence assessed by the GRADE approach assess.

For analysis of the safety of treatments, it is important to check their adverse effects. These were evaluated in only four [12,13,15,18] of the seven studies found. In two studies [13,15], no adverse effects were reported with the treatments performed. One study [18] reported three cases of gastric discomfort among participants who used penicillin. Comparing diclofenac and flurbiprofen versus topical spray benzydamine [12] found fewer adverse effects in the groups that used anti-inflammatory drugs in capsules. In these, cases of gastrointestinal disorders, easily reversed with gastric protectors, have been reported. The topical anesthetic generated oral numbness and alteration of the taste of participants in 11 of 12 participants. Despite the difference between the groups, there were wide confidence intervals and imprecise results to support this evidence.

The methodological quality assessment of the seven studies reveals that the majority present a high or unclear risk of bias to the domains analyzed. This fact suggests that the internal validity of the included clinical trials may be compromised and hinder the use of evidence in clinical decisions. None of the seven studies made clear how participants were randomized, which may influence the selection of participants for different treatments. If there is a failure in the random sequence generation, there is no same chance of the patient belonging to the different interventions. In three studies [13,15,18], the high risk of bias found about the participant’s blinding may suggest that participants were influenced by knowing which treatment was being performed. Additionally, the outcome assessors may have performed suggested ratings. All these factors together may have influenced the results.

It is essential to highlight that the limitations of the included studies and the lack of standardization of a gold standard treatment made it difficult to analyze the efficacy and safety of treatments for pericoronitis. This systematic review followed all the rigorous recommendations of the Cochrane Handbook of Systematic Reviews of Interventions and seemed to be the first systematic review about treatments for pericoronitis. Despite all the care adopted, this review has limitations. The clinical diversity and small sample size of the included studies was the greatest limitation found. Additionally, our search resulted in two papers of which we did not have access to the articles in full [8,9]. The authors were contacted; however, we did not get a reply to emails. We also found two ongoing studies that could provide relevant results when published and maybe modify this review’s results.

## 4. Materials and Methods

### 4.1. Study Design

This study followed the methodological recommendations of the Cochrane Handbook for Systematic Reviews of Interventions [21] and the PRISMA statement (Preferred Reporting Items for Systematic Reviews and Meta-Analyses) [22]. In addition, the review protocol was registered in the International Prospective Register of Systematic Reviews (PROSPERO) (CRD42020200637).

### 4.2. Eligibility Criteria

We have only included randomized clinical trials (RCTs) studies, with a parallel design involving adolescents and adults presenting pericoronitis. We have considered any noninvasive treatment such as saline irrigation, anti-inflammatory, antibiotic, photodynamic therapy, mouthwashes, among others. We have excluded studies with distinct co-treatments between different groups and studies with invasive treatments (third molar extraction).

The eligibility criteria were based on the PICO strategy, as follows:

- Population (P): Adolescents and adults (up to 12 years) presenting pericoronitis.
- Intervention (I): Noninvasive pericoronitis therapy.
- Comparison (C): Placebo, no intervention or different interventions compared to each other.
- Outcomes (O):

### 4.3. Primary Outcomes

- Pain relief (measured by validated scales, as Visual Analog Scale, among others) [23,24];
- Reduction of pro-inflammatory cytokines and increase of anti-inflammatory cytokines (measured by ELISA enzyme-linked immunosorbent assay (picograms/milliliters—pg/mL) [25];
- Microbiological assessment, measured by PCR analysis (polymerase chain reaction) [26] or by number of bacterial counts (colony-forming units—CFU) [27];
- Serious adverse events.

### 4.4. Secondary Outcomes

- Any adverse events, the proportion of participants with at least one adverse event during or subsequent treatment (for example, allergy);
- Oral health-related quality of life (OHQoL) (measured by valid questionnaires) [28];
- Trismus (measuring the inter-incisal distance between maxillary and mandibular using a caliper) [4];
- Recurrence of pericoronitis,
- Patient acceptability.

We considered all-time points reported by the RCTs, but we only pooled similar time points: short term (immediately after treatment to one month), intermediate-term (one to three months) and long term (more than three months).

### 4.5. Search Strategy

On 24 August 2021, we performed a sensitive search to identify studies that fulfilled our inclusion criteria without date, language, or publication status restrictions. The electronic search was developed in the following databases:

- The Cochrane Central Register of Controlled Trials—CENTRAL (via Wiley);
- MEDLINE (via PubMed);
- BBO (Bibliografia Brasileira de Odontologia—via Biblioteca Virtual em Saúde—BVS) Literatura Latino Americana em Ciências da Saúde e do Caribe—LILACS (via Biblioteca Virtual em Saúde—BVS);
- EMBASE (via Ovid).

Also, we performed a search for ongoing clinical trials on the following registration platforms: World Health Organization (WHO) International Clinical Trials Registry Platform (ICTPR) (apps.who.int/trialsearch, accessed on 1 January 2022) and Clinicaltrials.gov (www.clinicaltrials.gov, accessed on 1 January 2022). Grey literature was also screened via OpenGrey (www.opengrey.eu, accessed on 1 January 2022). We performed a hand search by contacting specialists in the field about any ongoing or awaiting publication studies. In addition, we have checked the list of references of relevant studies included in the systematic review and searched for abstracts of some specific dentistry conferences (e.g., International Association for Dental Research—IADR). The search strategies defined for each database are detailed in Table S3.

### 4.6. Selection of Studies and Data Collection Process

Two independent authors (ACRTH and TOS) selected titles and abstracts of the references retrieved by our search strategy using the Rayyan [29] software (https://rayyan.qcri.org, accessed on 1 January 2022). References classified as ‘potentially eligible’ were read in full to confirm their inclusion. Two independent authors (SKB and LJM) performed the data extraction process using a pre-established data extraction form. All discordance in selection and extraction processes were solved by consensus (EMS and ALCM). When needed, we contacted trial authors for additional information.

### 4.7. Methodological Quality Assessment

The risk of bias assessment was performed by two independent authors (ACRTH and ALCM), using the Cochrane Risk of Bias tool (RoB) [30] considering the following domains: random sequence generation, allocation concealment, blinding of participants and personnel, blinding of outcomes assessors, incomplete outcome data, selective reporting of outcomes and other potential sources of bias (e.g., baseline imbalances). A third author was consulted in case of discordances (SKB). The risk of bias for blinding and incomplete outcome data domains were planned to perform at the outcome level, according to (1) objective outcomes (serious adverse events) and (2) subjective outcomes (all other outcomes).

### 4.8. Data Synthesis and Analysis

The individual participants were considered as the unity of analysis. For the treatment effects estimate, we planned to calculate mean differences (MD) for continuous outcomes and risk ratio (RR) for dichotomous outcomes (considering a 95% confidence interval). When possible, treatment effects would be combined using a random effect model meta-analysis using the Review Manager 5.4.1 software [19]. Heterogeneity between studies was planned to be measured by Chi^2^ test, considering *p* > 0.1 as substantial heterogeneity and I2 statistics to measure the inconsistency between included studies (I2 > 50% means substantial heterogeneity) [21].

### 4.9. Subgroups Analysis

Subgroup analyses were planned for (a) different ages (adolescents vs. adults) and (b) different stages of pericoronitis (acute vs. chronic).

### 4.10. Publication Bias

Publication bias was planned to be investigated if a meta-analysis included more than 10 studies.

### 4.11. Assessment of the Certainty of the Evidence

We used the Grading of Recommendations, Assessment, Development and Evaluations (GRADE) [31] approach to assessing the certainty of the body of the evidence for all outcomes of the most clinically relevant comparison among treatments. The GRADE approach includes five domains: risk of bias, inconsistency, imprecision, indirectness, and publication bias. We specified the reasons for downgrading the certainty of the evidence. We created a Summary of Findings table (SoF table) using the GRADEpro GDT software [32].

## 5. Conclusions

Evidence of low methodological quality and high clinical diversity showed that there are still uncertainties to estimate the effect of the different interventions for pericoronitis. It is important to note that pericoronitis is an inflammation of the tissues around the crown. Until now, initial pericoronitis should be resolved with local irrigation and gently debridement. Antibiotics should be specially reserved for severe cases when systemic dissemination are present. Thus, the findings of this review are insufficient to support clinical decision making. It is necessary to consider the particularities of participants as well as the socioeconomic context and other related aspects for the choice of treatment. Future randomized clinical trials with methodological rigor, according to CONSORT (Consolidated Standards of Reporting Trials [19], with larger sample sizes, are essential to define better guidelines for clinical practice in the treatment of initial pericoronitis.

## Figures and Tables

**Table 1:** Main characteristics of seven full text randomized clinical trials on pericoronitis therapy.

Author/Year/Country	Participants	Interventions/Comparators	Outcomes of Interest	Follow up	Funding
Alalwani [12] 2019 Turkey	*n* = 60 29 male/31 female mean age 21.03 ± 1.99 (range 18 to 25 years) Mild pericoronitis	Group A: diclofenac 50 mg (1 capsule 8/8 h 7 d + placebo spray (4×)–6×/d 7 d) (*n* = 20) Group B: flurbiprofen 100 mg (1 capsule 3×/day for 7 d+ placebo spray) (*n* = 20) Group C: topical benzydamine (0.045 g, 30 mL oral spray + placebo capsule) (*n* = 20)	Quality of life (OHQoL questionnaire) Any adverse events	7 days	No financial support
Culhane [18] 1947 Not declared	*n* = 18 9 male/9 female mean age 23.3 years (range 21 to 29) Acute pericoronitis	Group A: An iodoform gauze drain, inserted daily to the base of the pocket (*n* = 6) Group B: 125,000 Oxford units of oral penicillin every three hours (*n* = 6) Group C: iodoform gauze drain was inserted daily to the base of the pocket + 125,000 Oxford units of oral penicillin every three hours (*n* = 6) Hot saline irrigations were prescribed every three hours in groups A and C	Any adverse events	6 days	Not declared
Elsade [15] 2020 Saudi Arabia	*n* = 59 participants male 33/female 26 Group A—17.4 ± 3.5/Group B—19.6 ± 5.1 Acute pericoronitis	Group A: Conventional treatment plus aPDT (methylene blue plus 660 nm) (*n* = 30) Group B: Conventional treatment (*n* = 29) Conventional treatment: conventional debridement (1×), warm saline water. Later, soft tissue swabbed with cotton with antiseptic, occlusion adjustment, ibuprofen 400 mg and OHC instruction	Pain Reduction of pro-inflammatory cytokines Microbiological assessment Any adverse events	7 and 14 days	Scientific Research at King Saud University (RG-1439-81).
Eroglu [13] 2018 Turkey	*n* = 40 male 19/female 21 mean age 22.97 (±3.4) years Pericoronitis-related lymphadenopathy	Group A: amoxicillin (1 g 12 h/12 h 7 days) (*n* = 20). Group B: amoxicillin +aPDT (indocyanine green + 810 nm) power of 0.3 W and sweeping technique, frequency 10,000 Hz, energy density 600 J/cm^2^, power density 15 W/cm^2^ (*n* = 20) Co-intervention: paracetamol 500 mg 8 h/8 h and chlorhexidine gluconate 4% mouthwash 3×/day for 7 d	Pain Any adverse events	7 days	Directorate of Scientific Research Projects of Yuzuncu Yil University
McGowan [17] 1977 England	*n* = 31 mean age 22.1 (16 to 29 years) 21 male/1 female Untreated severe acute pericoronitis	Group A: phenoxymethylpenicillin (250 mg, 1 tablet 4×/d for 5 days) (*n* = 13) Group B: Metronidazole (200 mg, 4×/day for 5 days) (*n* = 18) Co-intervention: Paracetamol (500 mg) as required for both groups	Trismus Any adverse events	7 days	Not declared
Shahakbari [16] 2014 Iran	*n* = 97 25.87 (±6.07) years male 34/female 63 Acute pericoronitis	Group A: green tea mouthrinse 5% 2×/d 7 d (*n* = 47) Group B: CHX mouthrinse 0.12% 2×/d 7 d (*n* = 50) Co-intervention: debridement + irrigation of the *operculum*. Amoxicillin (500 mg, 21 caps, 3×/d + optional analgesic (acetaminophen, 500 mg, 15 caps, 3×/d)	Pain Trismus	7 days	No financial support
Sezer [14] 2012 Turkey	*n* = 80 39 male/41 female Age range: 18–33 years Pericoronitis	Group A: laser placebo Group B: 808-nm diode (GaAlAs) laser 10 s, distance of 1 cm, continuous mode 0.25 W (*n* = 20) Group C: 660-nm diode laser 0.04 W, continuous mode, 60 s, 8 J/cm^2^ a distance of 1 cm (*n* = 20) Group D: 1064-nm Nd:YAG laser 10 s, distance of 1 cm, 0.25 W, frequency was 10 Hz, 8 J/cm^2^ (*n* = 20) Co-intervention: debridement and irrigation of pericoronal flap + 1000 mg of amoxicillin trihydrate/potassium clavulanate 12 h/12 h–7 d + 500 mg acetaminophen 8 h/8 h–5 d + rinsing 2×/day for 10 d with chlorhexidine 0.12%	Pain Quality of life (OHIP 14) Trismus	7 days	Not declared

RCT: Randomized clinical trial; *n*: number of participants; yrs: years; d: days, h: hours, x-times; OHQoL: oral health quality of life; CFU/mL Colony-forming units per millilitre; log: logarithm; CHX: Chlorhexidine; MMO: maximum mouth opening; OHC: oral health care; s: seconds; VAS: Visual analogue scale; g: grams; mg: milligrams; mL: microliters; caps: Capsules, h: Hour, TNF- Necrosis tumoral factor, IL- interleukin; GaAlAs—Gallio, Aluminium, arsenate; aPDT: antimicrobial photodynamic therapy; nm: nanometers; TNF: tumour necrosis factor; IL: interleukin; W: Watts; Hz: Hertz; cm: centimetres; J: Joules; cm^2^: square centimetres; mm: millimetres.

## Data Availability

Data reported in this study is available with the corresponding author, at a reasonable request.

## Supplementary Materials

The following supporting information can be downloaded at: https://www.mdpi.com/article/10.3390/antibiotics11010071/s1, Table S1: Risk of bias assessment—judgement details; Table S2: Summary of findings table (GRADE approach); Table S3: Search strategies (run on 24 August 2021).
