# Supplementary material for: Interventions for Early-Stage Pericoronitis: Systematic Review of Randomized Clinical Trials
Source: Antibiotics (Basel). 2022 Jan 8;11(1):71. doi: 10.3390/antibiotics11010071 (PMC8772688; doi:10.3390/antibiotics11010071)
Supplement: Supplementary file 1 [file antibiotics-11-00071-s001.zip › antibiotics-1527019-supplementary.pdf]

antibiotics

Review

Interventions for Early-Stage Pericoronitis: Systematic Review of Randomized Clinical Trials

Tânia Oppido Schalch [1], Ana Luiza Cabrera Martimbianco [2], Marcela Leticia Leal Gonçalves [2], Lara Jansiski Motta [1], Elaine Marcilio Santos [2], Rebecca Boltes Cecatto [1], Sandra Kalil Bussadori [1] and Anna Carolina Ratto Tempestini Horliana [1,*]

[1] University Nove de Julho (UNINOVE), São Paulo 01504-000, Brazil; taniaschalch@gmail.com (T.O.S.); larajmotta@uni9.pro.br (L.J.M.); rebeca.boltes@uni9.pro.br (R.B.C.); sandra.skb@gmail.com (S.K.B.)
[2] Universidade Metropolitana de Santos (UNIMES), São Paulo, Brazil; analuizacabrera@hotmail.com (A.L.C.M.); marcelalleal@hotmail.com (M.L.L.G.); elaine.marcilio@unimes.br (E.M.S.)
* Correspondence: annacrth@gmail.com; Tel.: +55-(11)-3385-9222

Supplementary Materials

Table S1. Risk of bias assessment – judgement details.

Study (author year)	Sequence generation	Allocation concealment	Blinding participants/ personnel	Blinding outcome assessors	Incomplete outcome	Selective reporting	Other bias
**Alalwani et al., 2019**	UNCLEAR Quote: "*Patients who met the inclusion criteria were randomly assigned to three groups*". Comment: Insufficient information on random sequence gerenation.	UNCLEAR Comment: Insufficient information on allocation concealment.	LOW Quote: "*The study medications were provided to the patients by the same clinic nurse, and the patients and the researchers were blinded to the study medication allocation.*"	LOW Quote: "*The study medications were provided to the patients by the same clinic nurse, and the patients and the researchers were blinded to the study medication allocation.*"	LOW No losses	HIGH Comment: The trial was registered (NCT03745599), but the paper did not report one important planned outcome (pain, measured by VAS).	LOW No other sources of bias
**Culhane et al., 1957**	UNCLEAR Quote: "*Eighteen patients selected at random from those applying for treatment*	UNCLEAR Comment: Insufficient information on	HIGH Comment: Blinding of participants and personnel was	HIGH Cooment: Blinding of outcome assessors was	LOW No losses	UNCLEAR Study protocol not available.	LOW No other sources of bias

	at the dental clinic comprised the group reported here." Comment: Insufficient information on random sequence gerenation.	allocation concealment.	unconfirmed and the interventions regimen were different. The subjective outcomes is likely to be influenced by this fact.	unconfirmed and the interventions regimen were different. The subjective outcomes is likely to be influenced by this fact.			
**Elsadek et al., 2020**	UNCLEAR Quote: "*Randomization technique was performed using block randomization. A total of 5 blocks of 6 participants were made for each group. The recruitment was done with the ratio of 1:1 and assigned letters 'X' and 'Y'*." Comment: Insufficient information on random sequence gerenation.	LOW Quote: "*Each recruited participant was given a code and masked in the envelope and identified with sequential numbers*." Comment: Allocation concealemnt adequately described.	HIGH Blinding of participants and personnel was unconfirmed and the interventions regimen were different. The subjective outcomes is likely to be influenced by this fact.	HIGH Blinding of outcome assessors was unconfirmed and the interventions regimen were different. The subjective outcomes is likely to be influenced by this fact.	LOW No substantial losses	LOW Quote: "registered in the research clinical trial database (GH062254)".	LOW No other sources of bias
**Eroglu et al., 2018**	UNCLEAR Quote: "The patients were randomly allocated to two groups" . Comment: Insufficient information on random sequence gerenation.	UNCLEAR Comment: Insufficient information on allocation concealment.	HIGH Comment: Blinding of participants and personnel was unconfirmed and the interventions regimen were different. The subjective outcomes is likely to be influenced by this fact.	HIGH Comment: Blinding of outcome assessors was unconfirmed and the interventions regimen were different. The subjective outcomes is likely to be influenced by this fact.	LOW No substantial losses	UNCLEAR Study protocol not available. Not found.	LOW No other sources of bias

**McGowan et al 1977**	UNCLEAR Comment: Insufficient information on random sequence gerenation.	LOW Quote: "*coded envelope containing either 20 x 200 mg tablets of metronidazole or 20 x 250 mg tablets of phenymethylpenic ilin*" Comment: Allocation concealemnt adequately described.	LOW Quote: "*Neither the clinician nor the patient knew wich drig was being taken*"	LOW Quote: "Neither the clinician nor the patient knew wich drig was being taken"	HIGH Comment: High rate of losses (27%)	UNCLEAR Comment: Study protocol not available. Not found.	LOW No other sources of bias
**Sezer et al., 2012**	UNCLEAR Quote: " *patients were randomly assigned to one of three LLLT groups or a placebo group*." Insufficient information on random sequence gerenation.	UNCLEAR Comment: Insufficient information on allocation concealment.	LOW Quote: "*The participants were blinded as to which treatment they received*."	LOW Quote: Measurements at all visits for a given subject were made by one calibrated examiner who was not involved in providing treatment during the study.	LOW Comment: No losses	UNCLEAR Study protocol not available. Not found.	LOW No other sources of bias
**Shahakbari et al.,2014**	UNCLEAR Comment: Insufficient information on random sequence gerenation.	UNCLEAR Comment: Insufficient information on allocation concealment.	LOW Quote: The patients had no idea of the mouthwash type pro- vided.	LOW Quote: Measurements were made by the second calibrated operator who had no idea to which group the patient had been assigned.	LOW Comment: No substantial losses (3%)	UNCLEAR Study protocol not available. Not found.	LOW No other sources of bias

Table S2. Summary of findings table (GRADE approach).

1. **Conventional treatment associated with antimicrobial photodynamic therapy (aPDT) *versus* conventional treatment**

2. **Patient or population: Pericoronitis**
3. **Setting: Ambulatory (outpatients)**
4. **Intervention: Conventional treatment protocol associated with antimicrobial photodynamic therapy (aPDT)**
5. **Comparison: Conventional treatment protocol**

6. **Outcomes**	7. **Anticipated absolute effects*** **(95% CI)**		8. **Relative effect (95% CI)**	9. **№ of participants (studies)**	10. **Certainty of the evidence (GRADE)**	11. Comments
	12. **Risk with Conventional treatment**	13. **Risk with Conventional treatment + aPDT**				
14. Pain assessed with: VAS Scale from: 0 to 10 follow-up: mean 14 days	15. The mean pain was **2.4** points	16. MD **0.4 points higher** (0.19 higher to 0.61 higher)	17. -	18. 59 (1 RCT)	19. ⊕◯◯◯ Very low[a,b]	20. The evidence is very uncertain about the effect of conventional treatment + aPDT on pain relief.
21. Reduction of pro-inflammatory cytokines TNFα assessed with: pg/mL follow-up: mean 14 days	22. The mean reduction of pro-inflammatory cytokines TNFα was **269** pg/mL	23. MD **128 pg/mL lower** (185.47 lower to 70.53 lower)	24. -	25. 59 (1 RCT)	26. ⊕◯◯◯ Very low[a,b]	27. The evidence is very uncertain about the effect of conventional treatment + aPDT on reducing pro-inflammatory cytokines TNFα.
28. Reduction of pro-inflammatory cytokines IL-6 29. assessed with: pg/mL follow-up: mean 14 days	30. The mean reduction of pro-inflammatory cytokines IL-6 was **47** pg/mL	31. MD **2 pg/mL lower** (10.72 lower to 6.72 higher)	32. -	33. 59 (1 RCT)	34. ⊕◯◯◯ Very low[a,b]	35. The evidence is very uncertain about the effect of conventional treatment + aPDT on reducing pro-inflammatory cytokines IL-6.
36. Microbiological count - Porphyromonas gingivalis 37. assessed with: CFU/mL follow-up: mean 14 days	38. The mean microbiological count - Porphyromonas gingivalis was **3.86** CFU/mL	39. MD **2.72 CFU/mL lower** (3.9 lower to 1.54 lower)	40. -	41. 59 (1 RCT)	42. ⊕⊕◯◯ Low[a,c]	43. Conventional treatment + aPDT may reduce/have little to no effect on microbiological count - Porphyromonas gingivalis.
44. Microbiological count - Tannerella forsythia 45. assessed with: CFU/mL follow-up: mean 14 days	46. The mean microbiological count - Tannerella forsythia was **3.7** CFU/mL	47. MD **0.98 CFU/mL lower** (1.76 lower to 0.2 lower)	48. -	49. 59 (1 RCT)	50. ⊕⊕◯◯ Low[a,c]	51. Conventional treatment + aPDT may reduce/have little to no effect on microbiological count - Tannerella forsythia.

52. ***The risk in the intervention group** (and its 95% confidence interval) is based on the assumed risk in the comparison group and the **relative effect** of the intervention (and its 95% CI).
CI: confidence interval; **MD:** mean difference

1. Conventional treatment associated with antimicrobial photodynamic therapy (aPDT) *versus* conventional treatment						
2. Patient or population: Pericoronitis						
3. Setting: Ambulatory (outpatients)						
4. Intervention: Conventional treatment protocol associated with antimicrobial photodynamic therapy (aPDT)						
5. Comparison: Conventional treatment protocol						
6. Outcomes	7. Anticipated absolute effects* (95% CI)		8. Relative effect (95% CI)	9. № of participants (studies)	10. Certainty of the evidence (GRADE)	11. Comments
	12. Risk with Conventional treatment	13. Risk with Conventional treatment + aPDT				
53. **GRADE Working Group grades of evidence**						
**High certainty:** we are very confident that the true effect lies close to that of the estimate of the effect. **Moderate certainty:** we are moderately confident in the effect estimate: the true effect is likely to be close to the estimate of the effect, but there is a possibility that it is substantially different. **Low certainty:** our confidence in the effect estimate is limited: the true effect may be substantially different from the estimate of the effect. **Very low certainty:** we have very little confidence in the effect estimate: the true effect is likely to be substantially different from the estimate of effect.						

Explanations: a. Downgraded two levels due to methodological limitations: unclear random sequence generation and high risk for blinding participants, personnel, and outcome assessors. b. Downgraded two levels due to imprecision (small sample size, wide confidence interval, single study). c. Downgraded one level due to imprecision (small sample size, single study).

Table S3. Search strategies (run on August 24th 2021).

Database	Search strategy	Results
**MEDLINE (via Pubmed)**	#1 "Pericoronitis"[Mesh] OR Pericoronitides	623
**CENTRAL**	#1 MeSH descriptor: [Pericoronitis] explode all trees	23
**LILACS/BBO (via BVS)**	#1 MH: Pericoronite OR Pericoronitis OR Pericoronitis OR Pericoronarite OR C07.465.714.258.771	74
**EMBASE (via Elsevier)**	#1 'gingiva disease'/exp AND pericoronitis AND [embase]/lim NOT ([embase]/lim AND [medline]/lim)	78
**Clinicaltrials.gov**	#1 Pericoronitis	6
**WHO/ICTRP**	#1 Pericoronitis	19
**OpenGrey**	#1 Pericoronitis	0
	**Total**	823
